# S-nitrosothiols, and other products of nitrate metabolism, are increased in multiple human blood compartments following ingestion of beetroot juice

**DOI:** 10.1016/j.redox.2021.101974

**Published:** 2021-04-16

**Authors:** Mohammed Abu-Alghayth, Anni Vanhatalo, Lee J. Wylie, Sinead TJ. McDonagh, Christopher Thompson, Stefan Kadach, Paul Kerr, Miranda J. Smallwood, Andrew M. Jones, Paul G. Winyard

**Affiliations:** a University of Exeter Medical School, College of Medicine and Health, St. Luke's Campus, University of Exeter, Heavitree Road, Exeter, EX1 2LU, UK; b Sport and Health Sciences, College of Life and Environmental Sciences, St. Luke's Campus, University of Exeter, Heavitree Road, Exeter, EX1 2LU, UK; c Royal Devon and Exeter NHS Foundation Trust, Exeter, EX1 2PD, UK

**Keywords:** Nitrate, Nitrite, S-nitrosothiols (RSNO), Plasma, Red blood cells (RBCs), Beetroot (BR)

## Abstract

Ingested inorganic nitrate (NO_3_⁻) has multiple effects in the human body including vasodilation, inhibition of platelet aggregation, and improved skeletal muscle function. The functional effects of oral NO_3_⁻ involve the *in vivo* reduction of NO_3_⁻ to nitrite (NO_2_⁻) and thence to nitric oxide (NO). However, the potential involvement of S-nitrosothiol (RSNO) formation is unclear. We hypothesised that the RSNO concentration ([RSNO]) in red blood cells (RBCs) and plasma is increased by NO_3_⁻-rich beetroot juice ingestion. In healthy human volunteers, we tested the effect of dietary supplementation with NO_3_⁻-rich beetroot juice (BR) or NO_3_⁻-depleted beetroot juice (placebo; PL) on [RSNO], [NO_3_⁻] and [NO_2_⁻] in RBCs, whole blood and plasma, as measured by ozone-based chemiluminescence. The median basal [RSNO] in plasma samples (n = 22) was 10 (5–13) nM (interquartile range in brackets). In comparison, the median values for basal [RSNO] in the corresponding RBC preparations (n = 19) and whole blood samples (n = 19) were higher (p < 0.001) than in plasma, being 40 (30–60) nM and 35 (25–80) nM, respectively. The median RBC [RSNO] in a separate cohort of healthy subjects (n = 5) was increased to 110 (93–125) nM after ingesting BR (12.8 mmol NO_3_⁻) compared to a corresponding baseline value of 25 (21–31) nM (Mann-Whitney test, p < 0.01). The median plasma [RSNO] in another cohort of healthy subjects (n = 14) was increased almost ten-fold to 104 (58–151) nM after BR supplementation (7 × 6.4 mmol of NO_3_⁻ over two days, p < 0.01) compared to PL. In conclusion, RBC and plasma [RSNO] are increased by BR ingestion. In addition to NO_2_⁻, RSNO may be involved in dietary NO_3_⁻ metabolism/actions.

## Highlights

• Human ingestion of NO_3_⁻-rich beetroot juice caused increased plasma S-nitrosothiol levels compared with baseline.
• Beetroot juice ingestion also caused increased S-nitrosothiol and NO_2_⁻ levels in red blood cells compared with baseline.
• RSNO formation may contribute to the physiological effects of dietary NO_3_⁻.

## Graphical abstract

**Figure:**
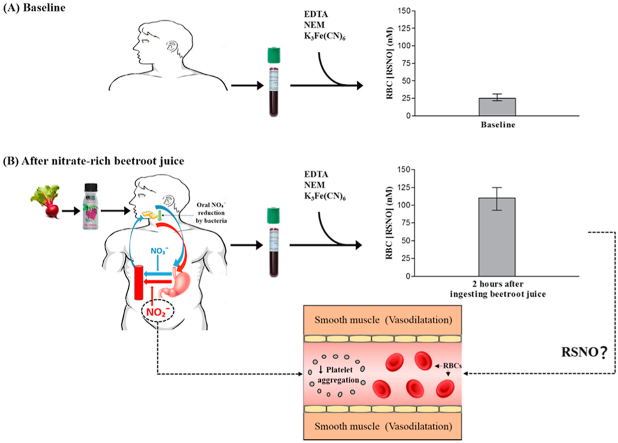

## 1 Introduction

Nitric oxide (NO) is a pivotal gaseous molecule that executes multiple functions in the human body, such as promoting vasodilation and lowering blood pressure [[1], [2], [3], [4]], inhibiting platelet aggregation [5] and enhancing exercise performance [[6], [7], [8]]. However, abnormalities in the bioavailability of NO and/or its reaction products [e.g., nitrate (NO_3_⁻), nitrite (NO_2_⁻), S-nitrosothiols (RSNO), N-nitrosamines (RNNO) and dinitrosyl iron complexes (DNICs)], have been linked to inflammation, hypertension, coagulation [9], diabetes mellitus [10] and reduced exercise tolerance [11]. NO is synthesised in endothelial cells by a reaction catalysed by three isozymes of nitric oxide synthase (NOS). These consist of neuronal (nNOS), endothelial (eNOS) and inducible (iNOS) isoforms of NOS. In the presence of oxygen (O₂) and reduced nicotinamide-adenine-dinucleotide phosphate (NADPH), which serve as co-substrates, these enzymes catalyse the conversion of l-arginine to l-citrulline and NO (the classical NO synthesis pathway) [12,13]. However, recent studies have demonstrated that, in humans, dietary NO_3_⁻ may also be converted to NO. This involves the entero-salivary circulation of NO_3_⁻, whereby NO_3_⁻ is absorbed in the stomach and small intestine, enters the blood circulation, and is then concentrated in saliva due to active uptake by the salivary glands. Secreted salivary NO_3_⁻ is converted to NO_2_⁻ in a reaction catalysed by bacterial NO_3_⁻ reductase (see below) in the oral cavity [[14], [15], [16]]. Swallowed NO_2_⁻ is then chemically reduced to NO under the acidic conditions of the stomach [17], as well as NO_2_⁻ being absorbed in the gastrointestinal tract and entering the bloodstream. When NO synthesis, catalysed by NOS, is impaired in human tissues, NO_2_⁻ is reduced to NO through several enzymatic and non-enzymatic pathways, particularly under hypoxic and acidic conditions, including biochemical reduction catalysed by deoxyhaemoglobin [18], deoxymyoglobin (which reduces NO_2_⁻ to NO at a rate 32 times faster than deoxyhaemoglobin) [19] and xanthine oxidase [20,21].

S-nitrosothiols (RSNO), also called thionitrites, include both low molecular weight RSNO, such as cysteine and glutathione, and high molecular weight RSNO such as S-nitrosoalbumin and S-nitrosohaemoglobin (SNO-Hb). RSNO are generated in tissues by the reaction of reactive nitrogen species (RNS) with thiol (RSH) groups. RSNO play an important role in vasodilation [22], inhibition of platelet aggregation [23,24], smooth muscle relaxation [25,26], and anti-inflammatory effects by protecting against ischemia/reperfusion injury [27]. RSNO are more stable than NO and are thought to act as NO donor molecules in the circulation. It has also been suggested that RSNO may be considered as a storage form for intravascular NO, which serves to regulate blood flow and oxygen delivery [28]. It is possible that RSNO enter the bloodstream after ingestion of green leafy vegetables and beetroot [29], which are rich sources of NO_3_⁻ [30]. In the NO_3_⁻ - NO_2_⁻ - NO pathway, NO_3_⁻ is rapidly reduced to NO_2_⁻ in the mouth by obligate and facultative anaerobic bacteria, resulting in a high salivary NO_2_⁻ concentration. Once swallowed, NO_2_⁻ in the stomach is exposed to a strongly acidic milieu (pH is ~1.5–2.0), thereby generating NO, nitrous acid and RNS, which promote RSNO formation [[31], [32], [33], [34], [35], [36]]. Gastric and plasma [RSNO] are increased after orally administered sodium NO_2_⁻ and NO_3_⁻ in hypertensive rats [35], plasma [RSNO] is increased after treatment with sodium nitrate (NaNO_3_) in mice with chronic ischemia [37], and gastric [RSNO] is increased after ingestion of inorganic NO_3_⁻ in humans [36,38]. However, it has been reported that plasma [RSNO] is not increased after NO_3_⁻ ingestion in humans [36,39].

RSNO, NO_3_⁻ and NO_2_⁻ concentrations have rarely been measured concurrently after ingestion of NO_3_⁻, and little is known about how these entities distribute themselves amongst the various blood compartments in humans. There is a large variation in the reported average baseline levels of RSNO in human blood compartments: e.g., the average plasma [RSNO] (reported as both mean and median values) varies between 6.3 nM and 7.19 μM in previous studies [28,[39], [40], [41], [42], [43], [44], [45], [46], [47], [48], [49], [50], [51]], many of which included only small sample sizes. In the present study, we hypothesised that the ingestion of inorganic NO_3_⁻ via beetroot juice increases the concentrations of RSNO in the red blood cells (RBCs) and plasma of healthy volunteers. We measured RSNO concentrations in different blood compartments in a relatively large number of human volunteers. In addition to measuring RSNO concentrations, we determined the concentrations of NO_3_⁻ and NO_2_⁻ in plasma, whole blood and RBCs, before and after NO_3_⁻-rich beetroot juice (BR) ingestion.

## 2 Materials, human samples and methods

### 2.1 Materials

NaNO_3_ was obtained from VWR international (Lutterworth, Leicestershire, UK). Sodium nitrite (NaNO_2_), N-ethylmaleimide (NEM), Nonidet™ P 40 Substitute, mercuric chloride (HgCl_2_) and 1 M hydrochloric acid (HCl) were obtained from Sigma-Aldrich Ltd. (Gillingham, UK). S-nitrosoglutathione (GSNO) was obtained from Enzo Life Sciences (Exeter, UK). Sodium iodide (NaI), potassium iodide (KI), iodine (I_2_), acetic acid (99.5%), ethylenediaminetetraacetic acid (EDTA), vanadium (III) chloride (VCl_3_), potassium ferricyanide, sulfanilamide and methanol were obtained from Fisher Scientific (Loughborough, Leicestershire, UK). Lithium-heparin tubes were obtained from Becton Dickinson UK Ltd. (Wokingham, UK). Cannulas were obtained from Becton Dickinson Insyte-WTM (Madrid, Spain). NO_3_⁻-rich beetroot juice (BR; beetroot juice containing 6.4 mmol of NO_3_⁻ per 70 ml; “Beet It Sport”) and NO_3_⁻-depleted beetroot juice (PL; beetroot juice containing 0.04 mmol of NO_3_⁻ per 70 ml) were obtained as gifts from James White Drinks Ltd. (Ipswich, UK). The reagents and buffers were made up in ultrapure water (18.2 MΩ/cm) unless otherwise indicated.

### 2.2 Human samples

The analysed samples were derived from four cohorts of volunteers. To measure the basal levels of RSNO in blood compartments, 22 adults (mean age ± standard deviation: 24 ± 3 years; 12 males and 10 females) were recruited. Seven adults (mean age: 23 ± 5 years; 4 males and 3 females) were recruited to study the effects of NO_3_⁻ ingestion on [NO_3_⁻] and [NO_2_⁻] in blood compartments. The effect of NO_3_⁻ ingestion on RBC [RSNO] in a separate group of 5 adult males (mean age: 26 ± 3 years) was investigated. To determine the effects of NO_3_⁻ ingestion on plasma [RSNO], 14 adults (mean age 25 ± 5 years; 10 males and 4 females) were recruited. All volunteers gave their written informed consent to participate in these studies which were approved by the Institutional Research Ethics Committee and conformed to the ethical principles of the Declaration of Helsinki. All subjects were healthy, not currently taking antibiotics, and none habitually used dietary supplements, mouthwash or smoked tobacco.

### 2.3 Methods and protocols

#### 2.3.1 Measurement of basal levels of RSNO in RBCs, whole blood and plasma

The concentrations of RSNO in RBCs, whole blood and plasma were determined at baseline (without BR). The healthy subjects (n = 22) attended the laboratory on a single occasion, not having consumed a dietary NO_3_⁻ bolus. Blood was collected, using a cannula inserted into the subject's antecubital vein, into two lithium-heparin vacutainers. The first collection tube contained final concentrations of ethylenediaminetetraacetic acid (EDTA) of 2.5 mM and NEM of 10 mM, respectively, to stabilise RSNO and block unreacted thiol groups in plasma samples [40,52,53]. The second tube contained EDTA, NEM and ferricyanide at final concentrations of 2.5, 10 and 10 mM, respectively. Ferricyanide stabilises SNO-Hb in RBC lysate samples by reacting immediately with haemoglobin to form methaemoglobin, hence hindering the auto-capture of NO by haem while preserving the SNO bond [48,51,[54], [55], [56], [57]]. A small (1.0 ml) whole blood sample was removed from the second tube and added immediately to 4.0 ml of hypotonic lysis solution containing EDTA, NEM and ferricyanide (at final concentrations of 2.5, 10 and 10 mM, respectively) to lyse the RBCs, before being stored in 1.5 ml Eppendorf tubes at −80 °C until analysis. All blood tubes were centrifuged at 3250 g for 10 min at 4 °C. The plasma was then removed from the first tube and stored in 1.5 ml Eppendorf tubes at −80 °C until analysis. Each RBC sample (1.0 ml) from each of the second tubes was immediately mixed with 4.0 ml of hypotonic lysis solution, to lyse the RBCs before then being stored in 1.5 ml Eppendorf tubes at −80 °C until analysis [58]. RSNO measurements were performed by treating biological samples (plasma, RBCs and whole blood) with 5% acidified sulfanilamide in 1 M HCl to eliminate nitrite [42]. Biological samples were also treated with a solution of 0.2% HgCl₂ in 5% acidified sulfanilamide and 1 M HCl to reduce RSNO to NO_2_⁻, as well as eliminating NO_2_⁻ [59]. Treatment consisted of mixing the biological sample with 5% acidified sulfanilamide with or without 0.2% HgCl₂ to give 1/10 dilutions of the reagents.

The concentrations of RSNO in these plasma, whole blood and RBC samples were measured by ozone-based chemiluminescence, a sensitive technique for the quantification of RNS. NO is obtained by chemically reducing NO_3_⁻/NO_2_⁻ or by cleaving NO from its parent compound(s) (e.g., RSNO) and that can be achieved by using a specific type of reducing solution for each measurement. For the measurement of RSNO, tri-iodide (I_3_⁻) reducing solution was present in the purge vessel at 60 °C to convert RSNO to NO [60]. I_3_⁻ solution was prepared by dissolving 2.0 g of potassium iodide (KI) and 1.3 g of I_2_ in 40 ml of ultrapure water. Then, 140 ml of acetic acid was added and mixed thoroughly for 30 min. The chemiluminescence reaction was based on the reaction of NO with ozone to produce the excited state of nitrogen dioxide (NO₂*) which returns to a ground state by releasing photons of light. The emitted light was detected by a photomultiplier tube housed within the NO analyser (Sievers NOA 280i; Analytix, Durham, UK) to generate an output voltage which was proportional to the NO concentration. The electrical signals (mV) were sent to a PC (NOAnalysis software v3.21) to facilitate analysis of the results [61,62]. Because of the low concentrations of total RSNO in human blood samples, a large purge vessel (50 ml) was used to ensure sufficient signal could be detected. The concentrations of RSNO in the samples were determined by using standard curves which were prepared with known concentration of S-nitrosoglutathione (GSNO, a low molecular weight RSNO) prior to analysis of the samples. A GSNO stock solution was prepared by adding 2.5 mg of GSNO to 1.0 ml of ultrapure water. The final concentration of the GSNO stock solution was determined by measuring its absorbance at 545 nm by using a Cary 300 UV–vis spectrophotometer. The molar extinction coefficient (*ε*) at 545 nm for GSNO is 17.2 M⁻^1^cm⁻^1^ [63]. Serial dilutions of known concentrations (0–1000 nM) of GSNO were used to construct a standard curve by treating the serially diluted GSNO solutions with 5% acidified sulfanilamide in 1 M HCl to eliminate NO_2_⁻. The mean correlation coefficient (r^2^) of GSNO standard curves was 0.9998. The between–batch coefficient of variation (CV) of the plasma RSNO assay was 10.7% (n = 5). To obtain the final chemiluminescence signal from RSNO, the signal of each biological sample treated with HgCl₂ was subtracted from the signal of the biological sample without HgCl₂.

#### 2.3.2 Assessment of the effects of BR ingestion on RBC RSNO concentrations

Before we started to measure [RSNO] in RBCs following BR ingestion, we wanted to check that when BR (2 × 70 ml; 12.8 mmol NO_3_⁻) was consumed by a second cohort of healthy volunteers (n = 7), there was an increase in the [NO_3_⁻] and [NO_2_⁻] in plasma (as previously reported; for example, see Refs. [[64], [65], [66], [67]]). We also measured [NO_3_⁻] and [NO_2_⁻] in whole blood and RBCs (Supplementary Figs. 1–2). Once we had confirmed that this amount of BR (12.8 mmol NO_3_⁻) did indeed cause an increase in [NO_3_⁻] and [NO_2_⁻], we measured [RSNO] in RBCs. NO_3_⁻-rich beetroot juice (2 × 70 ml; 12.8 mmol NO_3_⁻) was administered orally to a third cohort of healthy volunteers (n = 5). The participants were instructed to arrive at the laboratory for one visit. In the morning, venous blood was collected using a cannula inserted into the subject's antecubital vein, and blood was collected into lithium-heparin vacutainers containing EDTA, NEM and ferricyanide (as described above). Blood samples were collected at rest (baseline) and 2 h after consuming BR. The whole blood was centrifuged at 3250 g for 10 min at 4 °C, to separate the plasma and buffy coat from the packed RBCs. A small RBC sample (1.0 ml) was removed and added to 4.0 ml of hypotonic lysis solution containing EDTA, NEM and ferricyanide (at final concentrations of 2.5, 10 and 10 mM, respectively) to lyse the RBCs, before being stored at −80 °C until analysis.

#### 2.3.3 Determination of the effects of BR ingestion on plasma RSNO concentrations

BR or PL (7 × 70 ml) were administered orally to the fourth cohort of healthy volunteers (n = 14) to determine the effect of medium-term dietary supplementation with BR on [RSNO] in plasma. This study followed a double-blind, placebo controlled, crossover study design. The volunteers were instructed to arrive at the laboratory for three separate visits in a fully rested and hydrated state, at least 3 h postprandial and having avoided strenuous exercise in the 24 h preceding each visit. The blood samples were collected during the first visit at baseline (i.e., without any NO_3_⁻ supplementation before taking the samples), but at the second and third visits each of the volunteers had consumed 7 × 70 ml of BR (44.8 mmol NO_3_⁻) or PL over 48 h before collecting the blood samples. The protocol of this experiment was previously described in detail (see Ref. [68]). It involved the ingestion of a higher dose of NO_3_⁻ than in previous short-term BR supplementation experiments, resulting in plasma NO_2_⁻ rising by ∼800% in the BR group from pre-to post-donation [68]. In the previous published study [68], from which the presently analysed samples were derived, no adverse events were noted. The [RSNO] in the plasma samples was measured by ozone-based chemiluminescence as described above [62].

#### 2.3.4 Statistical analyses

All calculations were performed using Graph-Pad Prism software (Graph-Pad Software version 5.04). The data are presented as the median with interquartile ranges [IQR, 25% (lower; LQ) and 75% (upper; UQ)]. The Mann-Whitney *U* test was used to determine the statistical significance of differences between group median values. In all cases, p ≤ 0.05 was considered statistically significant and “n.s.” denotes “not statistically significant”. Spearman's correlation coefficient (r_s_) was used to explore significant relationships between the levels of [NO_3_⁻], [NO_2_⁻] and [RSNO] across the blood compartments and changes in the levels of [NO_3_⁻], [NO_2_⁻] and [RSNO] before and after ingesting BR. A Bonferroni correction was applied to adjust for the multiple tests against the same data set.

## 3 Results

### 3.1 Confirmation that BR ingestion resulted in a significant increase in plasma [NO_3_⁻] and [NO_2_⁻]

Typical examples of chemiluminescence time-traces when detecting NO_3_⁻ and NO_2_⁻, together with representative standard curves, are shown in Supplementary Fig. 1. As expected, the ingestion of BR containing 12.8 mmol of NO_3_⁻ resulted in statistically significant increases in the median plasma concentrations of both NO_3_⁻ and NO_2_⁻ compared with baseline and PL consumption (Supplementary Fig. 2).

### 3.2 Measurement of basal levels of RSNO in RBCs, whole blood and plasma

**Fig. 1:**
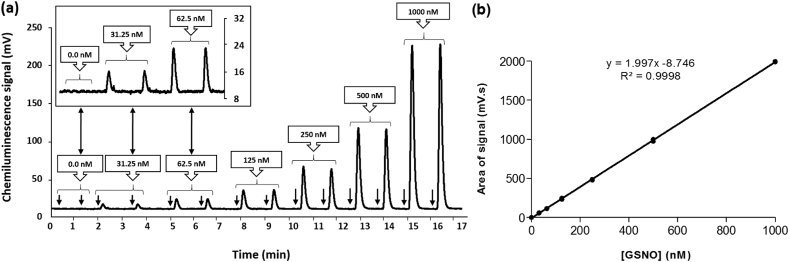
**Typical GSNO standard curve obtained from the depicted ozone-based chemiluminescence traces**. Panel (a): serial dilutions of a GSNO solution treated with a solution of 5% acidified sulfanilamide, were injected in duplicate into tri-iodide solution to obtain the chemiluminescence signal peaks which were quantified by calculating the area under the curve. The inset panel shows the traces from the lowest GSNO concentration, on an expanded y-axis scale for clarity. **↓** indicates the time-point at which the analysed sample (500 μl) was injected. Panel (b): an example of the resulting standard curve. The results shown are typical of those obtained in >20 experiments.

**Fig. 2:**
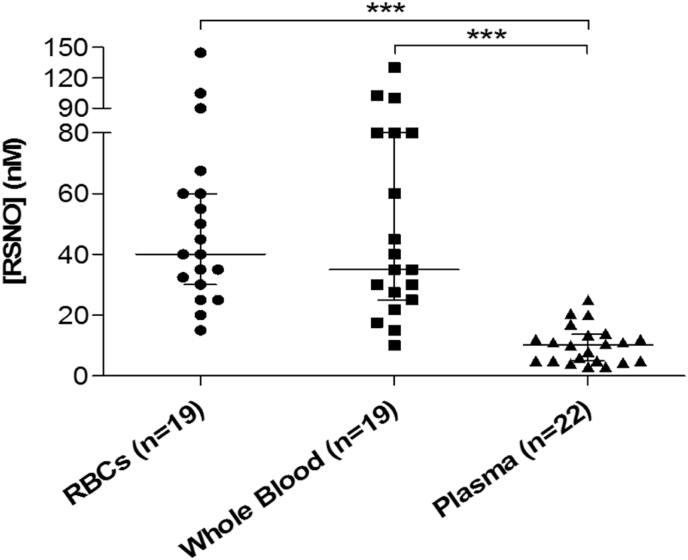
**Scatter plot showing the concentrations of total RSNO in plasma, RBCs and whole blood in healthy volunteers at baseline**. The graph shows that the median levels of total RSNO in healthy volunteers were higher (***p < 0.001) in RBCs and whole blood compared to plasma. The long horizontal bar represents the median value in each group, whilst the short horizontal bars represent the interquartile range.

**Fig. 3:**
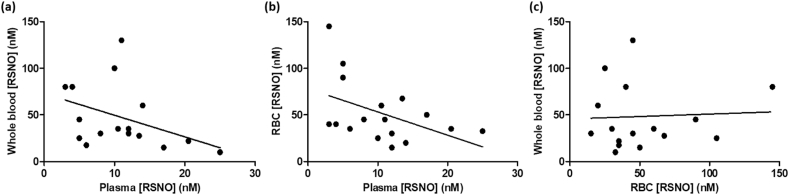
**Scatter plots showing the correlations of baseline total [RSNO] between blood compartments (plasma, whole blood and RBCs).** Panel (a) shows that the baseline [RSNO] in plasma was inversely correlated (r_s_ = − 0.53, p = 0.029) with whole blood [RSNO] from the same subjects. Panel (b) shows the observed trend towards a negative correlation between the baseline [RSNO] in plasma and in RBCs, but this was not statistically significant (r_s_ = − 0.44, p = 0.07). Panel (c) shows that the baseline [RSNO] in whole blood was not correlated (r_s_ = 0.001, p = 0.99) with RBC [RSNO]. The solid line indicates the estimated regression line. Spearman's rank correlation coefficient (r_s_) was calculated from n = 17 subjects.

We were able to confirm the expected effects of the reagents (5% sulfanilamide and 0.2% HgCl₂) on the observed chemiluminescence signals from the reagent-treated samples comprising the GSNO standard curve (Supplementary Fig. 3). A representative RSNO signal time-trace and standard curve is shown in Fig. 1. The median concentrations (with interquartile range in brackets) of total RSNO in healthy human volunteers at baseline (i.e., prior to any NO_3_⁻ supplementation) were 40 (30-60) nM, 35 (25-80) nM and 10 (5-13) nM in RBCs (n = 19), whole blood (n = 19) and plasma (n = 22), respectively (Fig. 2). The chemiluminescence assay of the total [RSNO] in these biological samples indicated that levels of total RSNO were significantly higher in RBCs and whole blood (p < 0.05) compared to matched plasma from the same individuals. Plasma [RSNO] at baseline exhibited a significant inverse correlation with whole blood [RSNO] (r_s_ = − 0.53, p < 0.05) from the same subjects (Fig. 3a). There was a trend towards a negative correlation between the baseline [RSNO] in plasma *versus* RBCs (Fig. 3b) (r_s_ = − 0.44, p = 0.07). In contrast, there was no correlation (r_s_ = 0.001) between [RSNO] in whole blood *versus* RBCs (Fig. 3c).

### 3.3 Assessment of the effects of BR ingestion on RBC RSNO concentrations

**Fig. 4:**
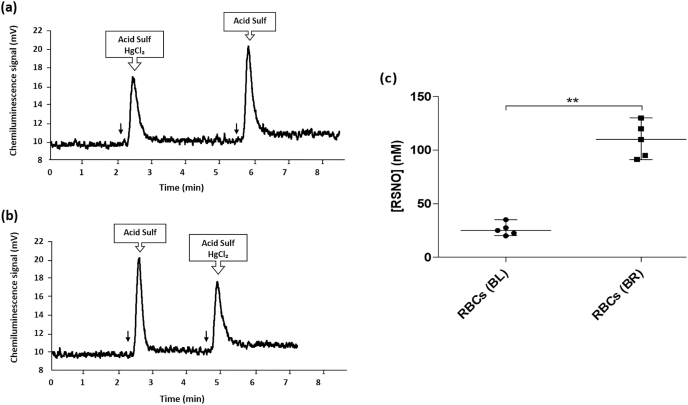
**Representative chemiluminescence time-traces forRBCRSNO detection, and a scatterplot showing the calculated concentrations of the total RSNO in RBCs of healthy volunteers.** Panels (a) and (b) show examples of chemiluminescence signal peaks from the ozone-based chemiluminescence during the measurement of RSNO in RBC samples from healthy volunteers, following sample injections (one with 5% sulfanilamide in 1 M HCl only (“Acid Sulf ") and another one with “Acid Sulf " and 0.2% HgCl₂) after individuals had ingested beetroot juice (BR) or at baseline (BL). ↓ indicates the time-point at which the analysed sample (500 μl) was injected. The final RSNO levels were obtained by subtracting the values of samples treated with HgCl₂ from those without HgCl₂. Panel (c) shows that the median concentration of RSNO in RBCs of healthy subjects was significantly higher (**, p < 0.01) after ingesting BR juice compared to the median baseline RSNO concentration without supplementation (n = 5).

We determined the effects of ingestion of 2 × 70 ml shots of concentrated BR on [NO_3_⁻] and [NO_2_⁻] in RBCs, whole blood and plasma. As expected from earlier studies (for example, Refs. [[64], [65], [66], [67]]), we found that the median RBC, whole blood and plasma [NO_3_⁻] and [NO_2_⁻] were significantly increased after ingesting BR (p < 0.05), compared to both baseline and PL consumption (Supplementary Fig. 2). In a separate group of healthy volunteers (n = 5), the median RBC [RSNO] was 25 (21-31) nM before ingesting BR and this was significantly increased to 110 (93–125) nM after ingesting BR (p < 0.01) (Fig. 4).

### 3.4 Determination of the effects of BR ingestion on plasma RSNO concentrations

**Fig. 5:**
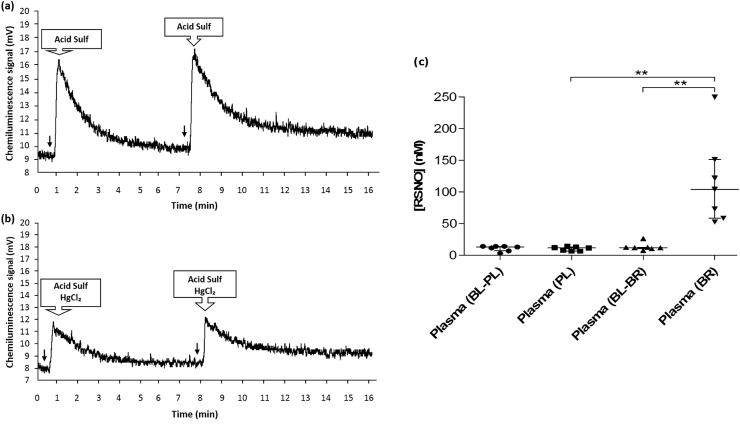
**Representative chemiluminescence signal traces for plasma RSNO detection, and a scatterplot showing the calculated concentrations of the total RSNO in the plasma of healthy volunteers at baseline, after ingesting the placebo juice, or after ingestingNO**_**3**_**⁻-richbeetroot juice.** The figure shows (in panels a and b) examples of the time courses of the chemiluminescence signal peaks when measuring the NO release from plasma RSNO in volunteers who had received NO_3_⁻-rich beetroot juice. Panel (a) shows typical results from injecting samples treated with 5% acidified sulfanilamide in 1 M HCl only (“Acid Sulf"), and panel (b) shows the signal traces for the same injected samples treated with 5% acidified sulfanilamide and 0.2% HgCl₂ in 1 M HCl (“Acid Sulf HgCl_2_″). The acid sulfanilamide and sulfanilamide/HgCl₂ solutions were diluted 1/10 when added to biological samples. In panels (a) and (b), ↓ indicates the time-point at which the analysed sample (500 μl) was injected. The final RSNO concentrations were calculated by subtracting the concentrations of the samples treated with HgCl₂ from the concentrations of the samples without HgCl₂. Panel (c) shows: the baseline levels of RSNO for a group of individuals (n=7) before administration of placebo (NO_3_⁻-depleted) beetroot juice (BL-PL), the levels of RSNO for the same group after ingesting placebo beetroot juice (PL), the baseline levels of RSNO for another group of individuals (n=7) before administration of NO_3_⁻-rich beetroot juice (BL-BR), and the levels of RSNO for the group after ingesting NO_3_⁻-rich beetroot juice (BR). Panel (c) shows that the median level of total RSNO in the plasma of healthy volunteers was significantly higher (**, p<0.01) after ingesting BR, compared to both the BL-BR and PL groups. However, there were no statistically significant differences between BL-PL, PL and BL-BR in relation to the median levels of total RSNO.

Total [RSNO] measurements were performed in 14 healthy volunteers before and after medium-term (48 h), repeated (7 × 70 ml), PL and BR supplementation. The median plasma [RSNO] of healthy volunteers at baseline and after ingesting PL and BR was 12 (11-13) nM, 11 (7-14) nM and 104 (58–151) nM, respectively (Fig. 5). The results indicated that the median total [RSNO] was significantly increased after ingesting BR (p < 0.05) compared to baseline and PL consumption. There was no statistically significant difference between median total [RSNO] in the BL group compared with the PL group. The values for [NO_3_⁻] and [NO_2_⁻] in this study of 14 healthy volunteers were previously reported [68]. There were no statistically significant correlations between the presently reported plasma [RSNO] and either [NO_3_⁻] or [NO_2_⁻].

## 4 Discussion

The main original findings of this study, which are consistent with our hypothesis, are that the RBC and plasma [RSNO] were increased after ingestion of BR, compared to baseline and PL in healthy volunteers. Plasma [NO_3_⁻] and [NO_2_⁻], and whole blood and RBC [NO_3_⁻] and [NO_2_⁻], were also increased after BR ingestion. It is possible that the increases in RBC and plasma [RSNO] and RBC [NO_2_⁻] may be involved in many of the diverse physiological effects reported following the ingestion of NO_3_⁻ because we have shown here that it is not solely plasma [NO_2_⁻] that is elevated following dietary NO_3_⁻ ingestion.

### 4.1 RBC, whole blood and plasma [RSNO] at baseline

Total RSNO in baseline human blood samples are present at low concentrations. In previous studies [28,39,[44], [45], [46], [47],^69^], the reported levels of total RSNO, in plasma of healthy individuals, have varied widely (between 6.3 and 7190 nM), possibly due to the use of different analytical methodologies. Early plasma RSNO measurements seemed to give relatively high concentrations, in the micromolar range. But, more recently, the reported values have become more consistent, at around 10 nM. In the present study, we used a large purge vessel (50 ml) to allow the injection of a larger volume of sample, to reduce foaming and obtain sufficient signal peak-to-noise ratios from the chemiluminescence traces. In earlier studies [45,48,50], only one type of RSNO species was measured in whole blood and RBC pellets, namely S-nitrosohaemoglobin (SNO-Hb). In these studies, RBC lysates from whole blood and RBC pellets were passed through Sephadex G25 size exclusion columns [45,48,50], to remove the small thiols and separate SNO-Hb, which has a relatively high molecular weight, in RBC lysates. However, in the present study all RSNO species in RBCs and whole blood were measured. Also, the levels of total RSNO in blood compartments were observed in a larger number of subjects (RBCs and whole blood, n = 19; plasma, n = 22), compared with earlier studies which mostly included a smaller number of subjects (between 3 and 10) [28,39,[42], [43], [44], [45], [46], [47], [48]].

In the present study, the basal [RSNO] was similar in RBCs (40 (30-60) nM) and whole blood (35 (25-80) nM); but these values were significantly higher than in matched plasma (10 (5-13) nM). The haematocrit is typically about 45% for human blood, which is consistent with our observation that the median concentration of RSNO in whole blood had a value which was intermediate between the median concentrations of RSNO in plasma and RBCs. Interestingly, there was a significant inverse correlation (r_s_ = − 0.53, p < 0.05) between [RSNO] in whole blood and [RSNO] in plasma (Fig. 3a). It seems possible that this inverse correlation might be explained, at least in part, by an inverse association between RBC [RSNO] and plasma [RSNO] (Fig. 3b), although this trend was not statistically significant. Whole blood [RSNO] showed no correlation with RBC [RSNO] (Fig. 3c). These relationships between [RSNO] in different blood compartments might suggest there is a ''fixed pool'' of RSNO in whole blood which can be distributed in different proportions between the RBC and plasma compartments. It has been reported that protein disulfide isomerase (PDI) can be S-nitrosated, such that PDI-SNO may be involved in the transport of intracellular NO to the cell surface and thence to the plasma [70]. Therefore, when the RBC [RSNO] is increased, the RBC PDI may be predicted to become S-nitrosated which could be involved in the transport of RSNO to the cell surface and then to the plasma. This mechanism might explain why a low [RSNO] in RBCs is associated with high [RSNO] in plasma and *vice versa*.

### 4.2 RSNO concentrations in RBCs and plasma following nitrate supplementation

We observed an increase in the levels of total RSNO in RBCs of healthy volunteers after ingesting BR, compared to baseline values. To our knowledge, this is the first study to report the concentrations of RSNO in RBCs from healthy humans after ingesting nitrate. One possible reason why earlier investigators did not observe an increase in blood RSNO after NO_3_⁻ ingestion might be because they did not analyse RBCs [36,39]. Our results also showed an increase in the levels of total RSNO in plasma of healthy volunteers after ingesting BR, which is in contrast to two previous studies [36,39]. This may be because a nitrate salt was used (pure potassium nitrate [36] or sodium nitrate [39]), whereas we used BR which may contain other components influencing the uptake and/or metabolism of nitrate [71,72]. For example, quercetin (a polyphenol) is one of the phytochemicals in BR [73,74]. Polyphenols may inhibit the formation of RSNO [[75], [76], [77]]. On the other hand, the ingestion of pure quercetin resulted in a significant increase in plasma RSNO in both normotensive volunteers [78] and 2K1C rats [79], potentially explaining why BR administration increases plasma RSNO, whilst pure sodium nitrate does not. Also, the placebo juice had no effect on RSNO levels in the present study, consistent with the effect of polyphenols in BR being synergistic with nitrate but absent when nitrate is removed from the juice.

In the Lundberg and Govoni study [39], another potential reason why an increase in [RSNO] in human plasma was not shown, after ingested nitrate, is that plasma [RSNO] was only monitored for 1.5 h after ingestion of sodium nitrate (10 mg/kg), whereas we monitored plasma [RSNO] for 2 h in the present study. In the Richardson et al. study [36] the amount of ingested NO_3_⁻ (2 mmol) was much smaller compared to our study (6.4 mmol) and may not have been physiologically meaningful. It has been shown that 3.5 mmol of dietary NO_3_⁻ (as beetroot juice), does not result in a blood pressure-lowering effect in healthy human volunteers with normal blood pressure [80,81].

The increased RBC and plasma RSNO concentrations observed in the present study could be due, at least in part, to the formation in the stomach of RSNO which are then absorbed. It has previously been reported that RSNO are formed in the human stomach after inorganic nitrate ingestion [36,38]. In peripheral blood, RSNO could be formed by the reaction of NO with thiyl radicals (RS·) or by a nitrosating species such as dinitrogen trioxide (N_2_O_3_) in the presence of RSH groups [52,82]. Other studies have also shown that the plasma concentration of total nitroso species (RXNO), which includes RSNO and N-nitrosamines (RNNO), was increased after ingestion of BR [83,84]. However, these two earlier studies did not distinguish RSNO from N-nitrosamines by using HgCl₂ to remove RSNO; these studies only used sulfanilamide to eliminate nitrite. Thus, the effects of BR ingestion on RXNO and RSNO should not be considered directly comparable. In the present study, the mercury-stable peaks in Fig. 4, Fig. 5b could be nitroso compound(s) that remained after incubation with HgCl_2_, such as N-nitrosamines, as suggested by previous studies [40,85]. Finally, dinitrosyl iron complexes (DNICs) cannot be excluded as contributors to the mercury-sensitive peaks in the same figures, as HgCl_2_ has been shown to accelerate the degradation of DNICs [86]. The future application to human blood samples of new ozone-based chemiluminescence methods for detecting DNICs may help to resolve this issue [87].

The levels of plasma NO_2_⁻ were increased following dietary NO_3_⁻ [5,18,[88], [89], [90]], and the *in vivo* conversion of NO_3_⁻ to NO_2_⁻ appears to be involved in many diverse physiological effects of dietary NO_3_⁻ such as vasodilation, lowering blood pressure and inhibition of platelet aggregation. The possibility remains, that after dietary NO_3_⁻ ingestion, these various physiological effects might also be mediated by the increased RBC and plasma [RSNO]. In previous studies, it has been shown that RSNO play a significant role in many positive physiological effects such as vasorelaxation and platelet inhibition [[22], [23], [24], [25], [26],^41^].

### 4.3 Conclusion

The median RSNO concentrations in plasma and RBCs from healthy volunteers were significantly higher after ingesting BR compared with PL and/or baseline concentrations. These results contrast with previous studies that have not shown increases in plasma [RSNO] and this may be because these studies used pure inorganic NO_3_⁻ and/or smaller doses of NO_3_⁻; the present study used BR, which may contain other components influencing the uptake/metabolism of NO_3_⁻. The observed inverse correlation between [RSNO] in whole blood versus [RSNO] in plasma at baseline, and a trend towards an inverse correlation between RBC [RSNO] and plasma [RSNO], suggests that the generated RSNO may be distributed in different proportions between the plasma and RBC compartments. These findings shed light on the distribution of different RNS between the various blood compartments and may help to improve the potential therapeutic strategies for use of NO_3_⁻ supplementation. Finally, measuring only plasma NO_3_⁻ and NO_2_⁻ may be insufficient to gain an understanding of the various physiological effects of dietary nitrate, which might be mediated – at least in part – by the increased RSNO in blood compartments.

## Declaration of competing interest

None.
