# Supplementary material for: S-nitrosothiols, and other products of nitrate metabolism, are increased in multiple human blood compartments following ingestion of beetroot juice
Source: Redox Biol. 2021 Apr 16;43:101974. doi: 10.1016/j.redox.2021.101974 (PMC8111767; doi:10.1016/j.redox.2021.101974)
Supplement: Multimedia component 1 [file mmc1.docx]

**Supplementary Material**

**for**

**Increased S-nitrosothiols, and other products of nitrate metabolism, in multiple human blood compartments following ingestion of beetroot juice.**

**Mohammed Abu-Alghayth^1^, Anni Vanhatalo^2^, Lee J Wylie^2^, Sinead TJ McDonagh^2^, Christopher Thompson^2^, Stefan Kadach^2^, Paul Kerr^3^, Miranda J Smallwood^1^, Andrew M Jones^2^ and Paul G Winyard^1^**

1. University of Exeter Medical School, College of Medicine and Health, St. Luke’s Campus, University of Exeter, Heavitree Road, Exeter, EX1 2LU, UK.
2. Sport and Health Sciences, College of Life and Environmental Sciences, St. Luke’s Campus, University of Exeter, Heavitree Road, Exeter, EX1 2LU, UK.
3. Royal Devon and Exeter NHS Foundation Trust, Exeter, EX1 2PD, UK.

**Table of Contents**

- **Supplementary Figure 1.** Typical examples of NO_3_ˉ **and** NO_2_ˉ **standard curves, as obtained from the presented representative examples of ozone-based chemiluminescence time-traces.**
- **Supplementary Figure 2.** Typical chemiluminescence time-traces for NO_3_ˉ and NO_2_ˉ detection, and the resulting scatter graphs which depict the quantification of the effect of nitrate supplementation on [NO_3_ˉ] and [NO_2_ˉ] in plasma, RBCs and whole blood of healthy volunteers.
- **Supplementary Figure 3.** Examples of typical chemiluminescence time-traces from S-nitrosoglutathione standard solutions.


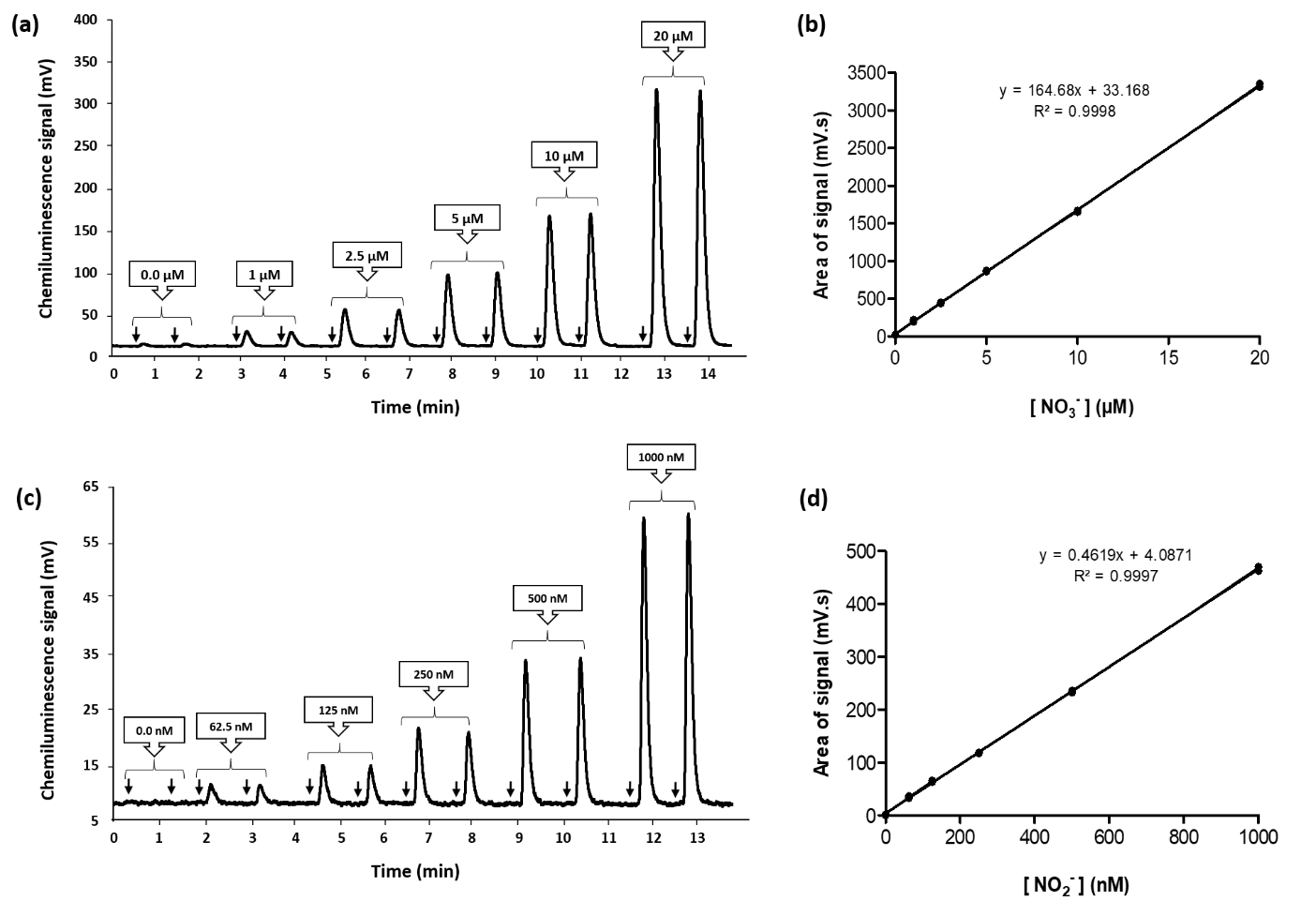


**Supplementary Fig. 1. Typical examples of** **NO_3_ˉ and NO_2_ˉ standard curves, as obtained from the presented representative examples of ozone-based chemiluminescence time-traces.** One of the most sensitive techniques for the quantification of NO_3_⁻/ NO_2_⁻ is ozone-based chemiluminescence, which allows the detection of low nanomolar quantities of NO, released from NO congeners, in several biological compartments. NO was obtained by chemically reducing NO_3_⁻/ NO_2_⁻ which was achieved by using a specific type of reducing solution for each measurement. For the measurement of NO_3_⁻, VCl_3_ in 1 M HCl (reducing agent) was placed in the purge vessel of an ozone-based chemiluminescence apparatus at 95 ^o^C, thereby facilitating the conversion of NO₃ˉ to NO (1-3). For the measurement of NO₂ˉ, NaI in acetic acid was placed in the purge vessel of an ozone-based chemiluminescence apparatus at 35 ^o^C, which facilitated the conversion of NO₂ˉ to NO (4, 5). The concentrations of NO_3_⁻ and NO_2_⁻ in the samples were determined by using standard curves which were prepared freshly with known concentrations of NO_3_⁻ and NO_2_⁻. Panel (a) shows an example of the results from serial dilutions of a NaNO_3_ solution, which were injected in duplicate into VCl_3_ in 1 M HCl (reducing agent) to obtain the chemiluminescence signal peaks which were quantified by calculating the area under the curve (NOAnalysis software v3.21, Analytix, UK). **↓** indicates the time-point at which the analysed sample (50 µl) was injected. Panel (b) shows an example of a NaNO_3_ standard curve, which was constructed to calculate the NO_3_⁻ concentration in the samples, expressed as μM (2, 3). Panel (c) shows an example of the results from serial dilutions of a NaNO_2_ solution, which were injected in duplicate into NaI in acetic acid (reducing agent) to obtain the chemiluminescence signal peaks which were quantified by calculating the area under the curve. **↓** indicates the time-point at which the analysed sample (100 µl) was injected. Panel (d) shows an example of a NaNO_2_ standard curve, which was constructed to calculate the NO_2_⁻ concentration in the samples, expressed as nM (6, 7). The mean correlation coefficients (R^2^) of the NO_3_⁻ and NO_2_⁻ standard curves were 0.9998 and 0.9997, respectively. The between–batch coefficients of variation (CVs) of the [NO_3_⁻] and [NO_2_⁻] assays were 2.6% (n=4) and 4.5% (n=4), respectively.


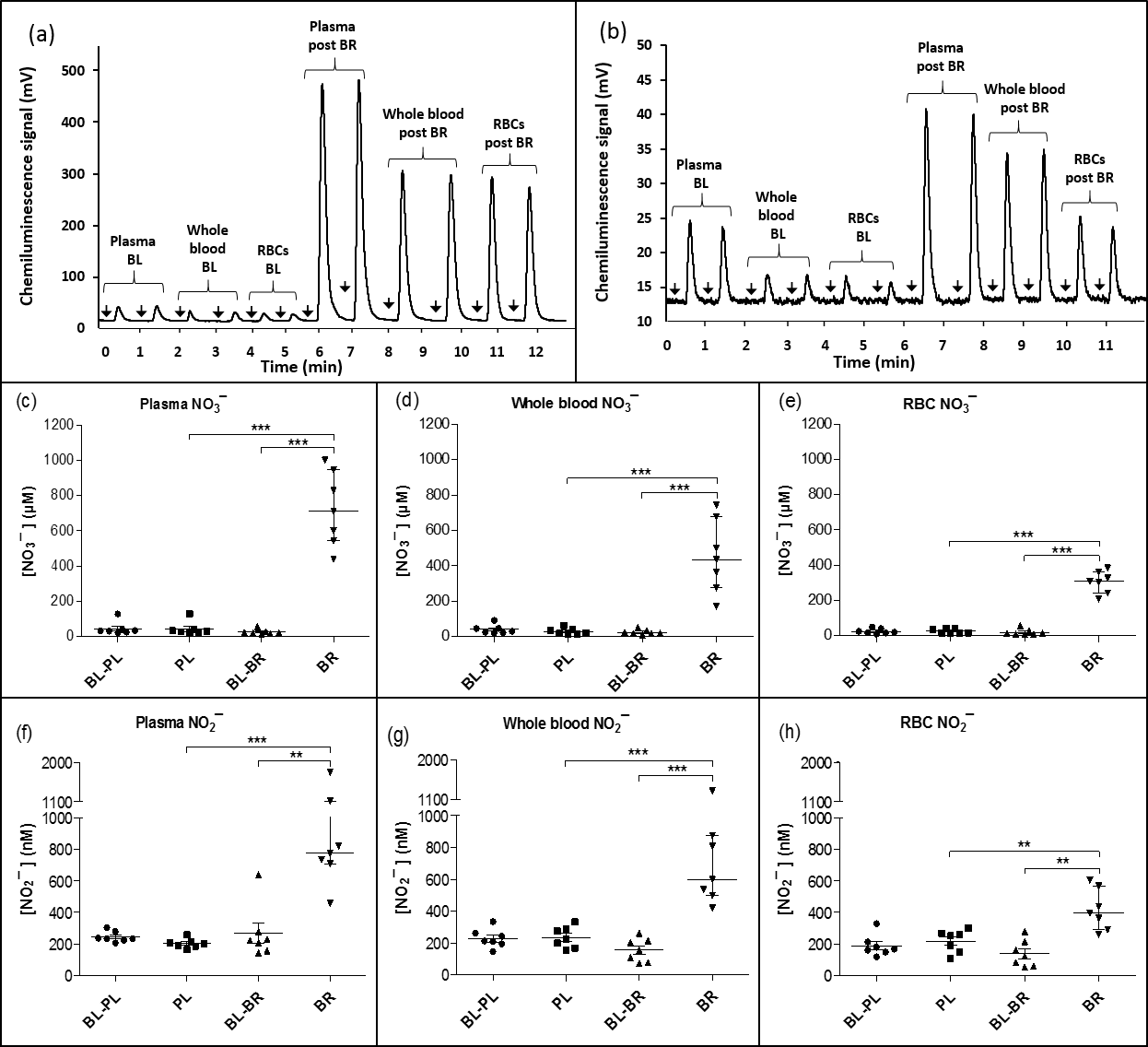


**Supplementary Fig 2.** **Typical chemiluminescence time-traces for NO_3_⁻ and NO_2_⁻ detection, and the resulting scatter graphs which depict the quantification of the effect of nitrate supplementation on [NO_3_⁻] and [NO_2_⁻] in plasma, RBCs and whole blood of healthy volunteers**. Panel (a) shows a typical ozone-based chemiluminescence time trace for [NO_3_⁻] measurement, and panel (b) shows a trace for [NO_2_⁻] measurement. The analysed samples consisted of plasma, whole blood and RBCs in healthy volunteers at BL and after ingesting placebo (NO_3_⁻-depleted) beetroot juice (PL) or NO_3_⁻-rich beetroot juice (BR). **↓** indicates the time-point at which the analysed sample (50 µl in panel (a) or 100 µl in panel (b)) was injected. The panels (c-d-e) show: the baseline levels of NO_3_⁻ for the group before administration of placebo (NO_3_⁻-depleted) beetroot juice (BL-PL), the levels of NO_3_⁻ for the group after ingesting placebo beetroot juice (PL), the baseline levels of NO_3_⁻ for the group before administration of NO_3_⁻-rich beetroot juice (BL-BR), and the levels of NO_3_⁻ for the group after ingesting NO_3_⁻-rich beetroot juice (BR). These results are shown for plasma (panel c), whole blood (panel d) and RBCs (panel e). The panels (f-g-h) show the baseline levels of NO_2_⁻ for the groups: BL-PL, PL, BL-BR, and BR, in plasma (panel f), whole blood (panel g) and RBCs (panel h). The panels (c-d-e-f-g-h) show that the median concentrations of plasma, whole blood and RBC NO_3_⁻ and NO_2_⁻ were significantly higher (**, P < 0.01; ***, P < 0.001) after ingesting BR compared to BL-BR and PL, but there were no significant differences between BL-BR and PL.

It has been shown in previous studies (1, 5, 8-17) that the concentrations of plasma NO_3_⁻ and NO_2_⁻ were increased after ingestion of NO_3_⁻, but the concentrations of NO_3_⁻ and NO_2_⁻ in RBCs or whole blood after ingesting NO_3_⁻ were not measured in these earlier studies. To determine the effects of nitrate ingestion on [NO_3_⁻] and [NO_2_⁻] in plasma, whole blood, and RBCs, the study participants (n=7) were instructed to arrive at the laboratory on two separate visits. On visit one, venous blood was collected in lithium-heparin tubes in the morning at rest (baseline) and 2 hours after consuming 2 × 70 ml of NO_3_⁻-rich beetroot juice (BR). On visit two, the same subjects followed the same protocol as in visit one, but they consumed 2 × 70 ml of NO_3_⁻-depleted beetroot juice (PL) instead of NO_3_⁻-rich beetroot juice (18). Whole blood samples were treated by adding a nitrite-preserving “stop” solution in the ratio 4:1 (v/v, sample/stop solution) to prevent nitrite destruction by oxy- and deoxy-haemoglobin (Hb) in the blood. The stop solution was prepared by mixing the following reagents: potassium ferricyanide (1.32 g), NEM (65 mg), Nonidet P-40 (0.5 ml), and ultrapure water (4.5 ml) (19-21). The sample was vortexed vigorously to allow complete lysis of cells, then frozen at ̶ 80 °C. Plasma and RBC samples were prepared from whole blood by centrifugation (3250 g, 4 °C, 5 min). The plasma was frozen immediately at ̶ 80 °C and in a similar way for RBC pellets as described previously for whole blood (19). Plasma, whole blood and RBC samples were deproteinised by aliquoting (300 µl) of the samples, which were then mixed with methanol in a 1:1 ratio (i.e., 300 µl of sample plus 300 µl of methanol). The samples were then vortexed and placed on ice for 30 min. The samples were then centrifuged at 13000 g for 5 min at 4 °C (19), and the supernatants analysed for NO_2_⁻ concentration. An aliquot 100 µl of each of the latter supernatants was removed for NO_3_⁻ analysis: the supernatant (100 µl) was diluted with ultrapure water (400 µl) and the samples were now ready for NO_3_⁻ analysis. The concentrations of NO_3_⁻ and NO_2_⁻ in these plasma, whole blood and RBC samples were measured using ozone-based chemiluminescence. NO_3_⁻ reduction by VCl_3_ chloride in 1 M HCl, and NO_2_⁻ reduction by NaI in acetic acid, were used to obtain chemiluminescence signal traces for [NO_3_⁻] and [NO_2_⁻] in plasma, whole blood and RBCs (as detailed in the legend to Supplementary Fig. 1).


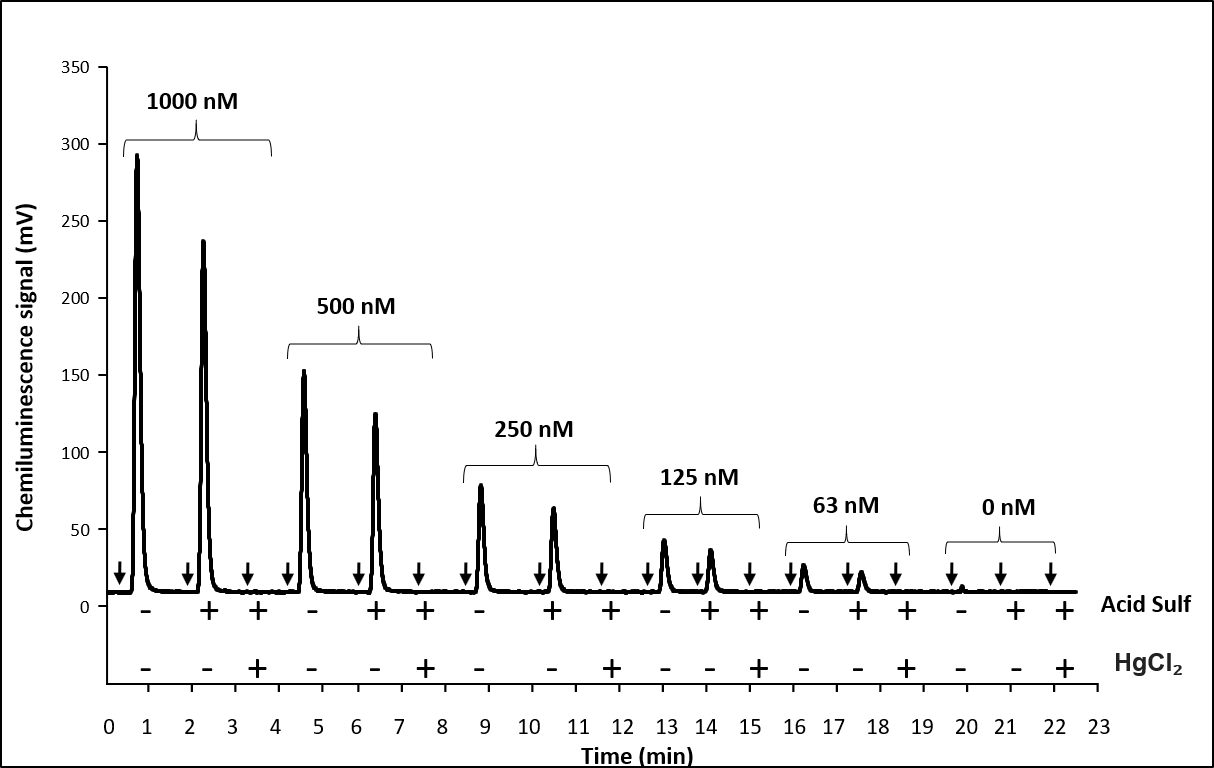


**Supplementary Fig. 3.** **Examples of typical chemiluminescence time-traces from S-nitrosoglutathione standard solutions**. There were three injections of each S-nitrosoglutathione (GSNO) standard, in which the first injected solution was untreated ( ̋– –̏ ), the second injected solution was treated with 5% acidified sulfanilamide (″Acid Sulf‶) in 1 M HCl ( ̋**+** –**̏** ), and the third injected solution had been treated with 5% acidified sulfanilamide and 0.2% HgCl₂ in 1 M HCl ( ̋**+ +̏** ). Note that there were some regions of the trace where no peaks were detectable after the sample injection. **↓** indicates the time-point point at which the analysed sample (500 µl) was injected.

**REFERENCES**

1. Lundberg JO, Govoni M. Inorganic nitrate is a possible source for systemic generation of nitric oxide. Free Radic Biol Med 2004;37(3):395–400. Available from: https://doi.org/10.1016/j.freeradbiomed.2004.04.027

2. Moriel P, Pereira IRO, Bertolami MC, Abdalla DSP. Is ceruloplasmin an important catalyst for S-nitrosothiol generation in hypercholesterolemia? Free Radic Biol Med 2000;30(3):318–26. Available from: https://doi.org/10.1016/S0891-5849(00)00467-6

3. Smárason A, Allman KG, Young D, Redman CWG. Elevated levels of serum nitrate, a stable end product of nitric oxide, in women with pre-eclampsia. BJOG: An Int J Obstet Gynaecol 1997;104(5):538–43. Available from: https://doi.org/10.1111/j.1471-0528.1997.tb11528.x

4. Garside C. A chemiluminescent technique for the determination of nanomolar concentrations of nitrate and nitrite in seawater. Mar Chem 1982;11:159–67. Available from: https://doi.org/10.1016/0304-4203(82)90039-1

5. Vanhatalo A, Bailey SJ, Blackwell JR, DiMenna FJ, Pavey TG, Wilkerson DP, et al. Acute and chronic effects of dietary nitrate supplementation on blood pressure and the physiological responses to moderate-intensity and incremental exercise. Am J Physiol Regul Integr Comp Physiol 2010;299:R1121–31. Available from: https://doi.org/10.1152/ajpregu.00206.2010

6. Ewing JF, Janero DR. Specific S-nitrosothiol (thionitrite) quantification as solution nitrite after vanadium(III) reduction and ozone-chemiluminescent detection. Free Radic Biol Med 1998;25(4–5):621–8. Available from: https://doi.org/10.1016/S0891-5849(98)00083-5

7. Piknova B, Park JW, Cassel KS, Gilliard CN, Schechter AN. Measuring nitrite and nitrate, metabolites in the nitric oxide pathway, in biological materials using the chemiluminescence method. J Vis Exp 2016;118:1–7. Available from: http://www.jove.com/video/54879

8. Larsen FJ, Weitzberg E, Lundberg JO, Ekblom B. Effects of dietary nitrate on oxygen cost during exercise. Acta Physiol 2007;191(1):59–66. Available from: https://doi.org/10.1111/j.1748-1716.2007.01713.

9. McDonagh STJ, Vanhatalo A, Fulford J, Wylie LJ, Bailey SJ, Jones AM. Dietary nitrate supplementation attenuates the reduction in exercise tolerance following blood donation. Am J Physiol - Hear Circ Physiol 2016;311(6):H1520–9. Available from: http://ajpheart.physiology.org/lookup/doi/10.1152/ajpheart.00451.2016

10. Bailey SJ, Fulford J, Vanhatalo A, Winyard PG, Blackwell JR, DiMenna FJ, et al. Dietary nitrate supplementation enhances muscle contractile efficiency during knee-extensor exercise in humans. J Appl Physiol 2010;109(1):135–48. Available from: http://jap.physiology.org/content/109/1/135

11. Wylie LJ, Mohr M, Krustrup P, Jackman SR, Ermdis G, Kelly J, et al. Dietary nitrate supplementation improves team sport-specific intense intermittent exercise performance. Eur J Appl Physiol 2013;113(7):1673–84. Available from: https://link.springer.com/article/10.1007/s00421-013-2589-8.

12. Bailey SJ, Winyard PG, Vanhatalo A, Blackwell JR, DiMenna FJ, Wilkerson DP, et al. Dietary nitrate supplementation reduces the O_2_ cost of low-intensity exercise and enhances tolerance to high-intensity exercise in humans. J Appl Physiol 2009;107(4):1144–55. Available from: https://doi.org/10.1152/japplphysiol.00722.200

13. Larsen FJ, Weitzberg E, Lundberg JO, Ekblom B. Dietary nitrate reduces maximal oxygen consumption while maintaining work performance in maximal exercise. Free Radic Biol Med 2010;48(2):342–7. Available from: http://dx.doi.org/10.1016/j.freeradbiomed.2009.11.006

14. Larsen FJ, Schiffer TA, Borniquel S, Sahlin K, Ekblom B, Lundberg JO, et al. Dietary inorganic nitrate improves mitochondrial efficiency in humans. Cell Metab 2011;13(2):149–59. Available from: https://doi.org/10.1016/j.cmet.2011.01.004

15. Lansley KE, Winyard PG, Bailey SJ, Vanhatalo A, Wilkerson DP, Blackwell JR, et al. Acute dietary nitrate supplementation improves cycling time trial performance. Med Sci Sports Exerc 2011;43(6):1125–31. Available from: https://pubmed.ncbi.nlm.nih.gov/21471821/

16. Lansley KE, Winyard PG, Fulford J, Vanhatalo A, Bailey SJ, Blackwell JR, et al. Dietary nitrate supplementation reduces the O_2_ cost of walking and running: a placebo-controlled study. J Appl Physiol 2011;110(3):591. Available from: http://jap.physiology.org/content/110/3/591.short

17. Wylie LJ, Kelly J, Bailey SJ, Blackwell JR, Skiba PF, Winyard PG, et al. Beetroot juice and exercise: pharmacodynamic and dose-response relationships. J Appl Physiol 2013;115(3):325–36. Available from: http://jap.physiology.org/cgi/doi/10.1152/japplphysiol.00372.2013

18. Gilchrist M, Winyard PG, Fulford J, Anning C, Shore AC, Benjamin N. Dietary nitrate supplementation improves reaction time in type 2 diabetes: Development and application of a novel nitrate-depleted beetroot juice placebo. Nitric Oxide - Biol Chem 2014;40:67–74. Available from: http://dx.doi.org/10.1016/j.niox.2014.05.003

19. Piknova B, Schechter AN. Measurement of nitrite in blood samples using the ferricyanide-based hemoglobin oxidation assay. Methods Mol Biol (Nitric Oxide) 2011;704:39–56. Available from: https://link.springer.com/protocol/10.1007/978-1-61737-964-2_4

20. Pelletier MM, Kleinbongard P, Ringwood L, Hito R, Hunter CJ, Schechter AN, et al. The measurement of blood and plasma nitrite by chemiluminescence: Pitfalls and solutions. Free Radic Biol Med 2006;41(4):541–8. Available from: https://doi.org/10.1016/j.freeradbiomed.2006.05.001

21. Dejam A, Hunter CJ, Pelletier MM, Hsu LL, Machado RF, Shiva S, et al. Erythrocytes are the major intravascular storage sites of nitrite in human blood. Blood 2005;106(2):734–40. Available from: https://doi.org/10.1182/blood-2005-02-0567
